# Supplementary material for: Identification of photoperiod-induced specific miRNAs in the adrenal glands of Sunite sheep (Ovis aries)
Source: Front Vet Sci. 2022 Jul 22;9:888207. doi: 10.3389/fvets.2022.888207 (PMC9354845; doi:10.3389/fvets.2022.888207)
Supplement: Supplementary file 1 [file Data_Sheet_1.ZIP › Supplementary file/miRDeep2 file.zip!/miRDeep2 file/Know_Structure/SPLP42A3/oar-mir-412.pdf]

miRBase precursor : oar-mir-412
Total read count : 665
oar-miR-412-3p read count : 650
oar-miR-412-5p read count : 7
remaining reads : 8

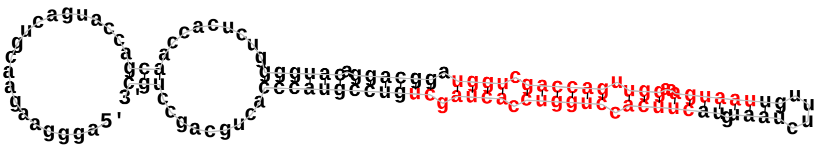

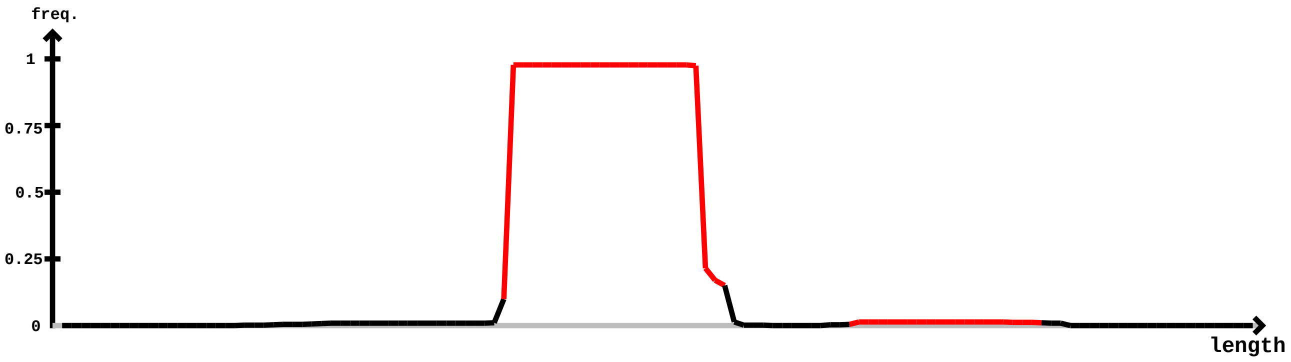

oar-miR-412-5p

oar-miR-412-3p

```
5'- agggaagaacgucaguaccagcaaccacucugggguacaggacggauggucgaccaguuggaaaguaauuguuucuaauguacuucaccugguccacuagcuguccguacccacugcagccugc   -3'    exp
    ..........(.((((..(((........)))((((((.((((((.((((.((((((.(((..((((((((....)))).))))))).)))))).)))).))))))))))))))))).......   reads   mm
    ..................cagcaaccacucugggguacaggacgg...............................................................................   1       0
    .....................caaccacucugggguacaggacg................................................................................   1       0
    ......................aaccacucugggguacaggacgg...............................................................................   1       0
    .........................cacucugggguacaggacgga..............................................................................   1       0
    ..........................acucugggguacaggacgga..............................................................................   1       0
    ...........................cucugggguacaggacgga..............................................................................   1       0
    ............................................gauggucgaccaguuggaaagu..........................................................   2       0
    .............................................auggucgaccaguuggaaagu..........................................................   56      0
    .............................................auggucgaccaguuggaaagua.........................................................   2       0
    .............................................auggucgaccaguuggaaaguaa........................................................   1       0
    .............................................auggucgaccaguuggaaaguaau.......................................................   1       0
    .............................................auggucgaccaguuggaaaguaauu......................................................   1       0
    ..............................................uggucgaccaguuggaaag...........................................................   2       0
    ..............................................uggucgaccaguuggaaagu..........................................................   447     0
    ..............................................uggucgaccaguuggaaagua.........................................................   28      0
    ..............................................uggucgaccaguuggaaaguaa........................................................   12      0
    ..............................................uggucgaccaguuggaaaguaau.......................................................   90      0
    ..............................................uggucgaccaguuggaaaguaauu......................................................   7       0
    ..............................................uggucgaccaguuggaaaguaauuguu...................................................   1       0
    ...............................................................................guacuucaccugguccacu..........................   1       0
    ...............................................................................guacuucaccugguccacuagc.......................   1       0
    .................................................................................acuucaccugguccacuagcu......................   1       0
    ..................................................................................cuucaccugguccacuagcugu....................   6       0
```
